# Study of Soluble HLA-G in Congenital Human Cytomegalovirus Infection

**DOI:** 10.1155/2016/3890306

**Published:** 2016-09-06

**Authors:** Roberta Rizzo, Liliana Gabrielli, Daria Bortolotti, Valentina Gentili, Giulia Piccirilli, Angela Chiereghin, Claudia Pavia, Silvia Bolzani, Brunella Guerra, Giuliana Simonazzi, Francesca Cervi, Maria Grazia Capretti, Enrico Fainardi, Dario Di Luca, Maria Paola Landini, Tiziana Lazzarotto

**Affiliations:** ^1^Department of Medical Sciences, Section of Microbiology and Medical Genetics, University of Ferrara, Ferrara, Italy; ^2^Operative Unit of Clinical Microbiology, St. Orsola-Malpighi University Hospital, Bologna, Italy; ^3^Operative Unit of Obstetrics and Prenatal Medicine, Department of Medical and Surgical Sciences, St. Orsola-Malpighi University Hospital, University of Bologna, Bologna, Italy; ^4^Operative Unit of Neonatology, St. Orsola-Malpighi University Hospital, Bologna, Italy; ^5^Operative Unit of Neuroradiology, Careggi University Hospital, Firenze, Italy; ^6^Operative Unit of Clinical Microbiology, Department of Specialised, Experimental, and Diagnostic Medicine, St. Orsola-Malpighi University Hospital, University of Bologna, Bologna, Italy

## Abstract

Human leukocyte antigen-G (HLA-G) is a nonclassical HLA class I antigen that is expressed during pregnancy contributing to maternal-fetal tolerance. HLA-G can be expressed as membrane-bound and soluble forms. HLA-G expression increases strongly during viral infections such as congenital human cytomegalovirus (HCMV) infections, with functional consequences in immunoregulation. In this work we investigated the expression of soluble (s)HLA-G and beta-2 microglobulin (component of HLA) molecules in correlation with the risk of transmission and severity of congenital HCMV infection. We analyzed 182 blood samples from 130 pregnant women and 52 nonpregnant women and 56 amniotic fluid samples from women experiencing primary HCMV infection. The median levels of sHLA-G in maternal serum of women with primary HCMV infection were higher in comparison with nonprimary and uninfected pregnant women (*p* < 0.001). AF from HCMV symptomatic fetuses presented higher sHLA-G levels in comparison with infected asymptomatic fetuses (*p* < 0.001), presence of HLA-G free-heavy chain, and a concentration gradient from amniotic fluid to maternal blood. No significant statistical difference of beta-2 microglobulin median levels was observed between all different groups. Our results suggest the determination of sHLA-G molecules in both maternal blood and amniotic fluid as a promising biomarker of diagnosis of maternal HCMV primary infection and fetal HCMV disease.

## 1. Introduction

Human cytomegalovirus (HCMV) is the most common cause of intrauterine infection, occurring in 0.3% to 2.3% of births [1]. HCMV intrauterine transmission is more common after primary infection (30–40% of probability) than after nonprimary infection (1%) [2, 3]. Nevertheless, it was estimated that, for all population seroprevalences, nonprimary maternal infections are responsible for the majority of congenital CMV infections [4].

Ten to fifteen percent of congenitally infected infants of primarily infected women will have symptoms at birth and around 10% of them will not survive. Moreover, 70–80% of surviving babies will suffer delayed sequelae such as sensorineural hearing loss, delay of psychomotor development, and visual impairment [5]. Most congenital infected infants (85–90%) have no symptoms at birth, but 8% to 15% of them will develop delayed injury [3, 5].

The fetal compartment can be studied by invasive (amniocentesis) and noninvasive (ultrasound examination) techniques [6]. Ultrasonographic findings are helpful but not diagnostic findings since HCMV has features in common with other intrauterine infections and its sensitivity is poor [7]. HCMV detection in amniotic fluid with virus isolation and/or Real-Time PCR is useful for prenatal diagnosis of fetal infection, due to its high sensitivity and specificity [8–10]. There is still a need for reliable prognostic factors for the outcome of HCMV fetal infection.

HCMV can modulate the expression and/or function of human leukocyte antigens (HLA), by encoding proteins to detain and destroy the expression of HLA molecules on the surface of infected cells, or selectively upregulate specific HLA class I molecules binding to immune cell inhibitory receptors [11]. In this scenario, there is an interesting nonclassical HLA class I antigen, HLA-G, characterized by low allelic polymorphism, restricted tissue distribution, and alternative mRNA splicing which creates different isoforms, 4 membrane-bound (HLA-G1–G4) and 3 soluble (HLA-G5–G7) [12]. In addition, the HLA-G1 isoform can produce a soluble form called sHLA-G1, derived from membrane proteolytic shedding [13]. HLA-G is expressed at the maternal-fetal interface, on surface of trophoblasts [12], and the concentrations of soluble (s)HLA-G increase in the plasma samples of pregnant women during the first trimester of pregnancy [14]. HCMV infection modifies HLA-G expression in tissues and immune cells, with a downmodulation in infected cytotrophoblasts [15] and upregulation in infected peripheral blood cells [16]. Specific HCMV proteins modify HLA-G expression interacting with the HLA-G promoter, and affecting mRNA stability, protein translation, and the secretory pathway [17–19]. The increase in HLA-G expression is suggested as a mechanism for virus immune escape, due to the immune-inhibitory functions of HLA-G. Finally, it has been observed that another important component of HLA class I molecules, beta-2 microglobulin (b2M), has a diagnostic efficacy for differentiating symptomatic from asymptomatic HCMV congenital infection [20].

In order to explore the possible role of HLA-G molecules in congenital HCMV infection, we analyzed maternal and fetal sHLA-G and b2M expression in correlation with the risk of transmission and severity of HCMV infection.

## 2. Materials and Methods

### 2.1. Subjects

The study analyzed the serum samples of a cohort of 130 pregnant women who were referred to the Maternal-Fetal Medicine Unit, St. Orsola-Malpighi University Hospital, Bologna, between 2006 and 2011 for suspected primary maternal HCMV infection. Maternal primary HCMV infection was assessed at the Virology Unit of the same University Hospital. Written informed consent for the studies was obtained from all patients according to the protocol approved by the Scientific Ethical Committee of the Ferrara and Bologna Universities.

The women, aged between 18 and 40 years, were in the first or second trimester of pregnancy. They presented no previous autoimmune and inflammatory diseases and they were not on any anti-inflammatory or immune-modulatory drugs or hyperimmune globulin.

Primary infection was diagnosed based on clinical and laboratory history and HCMV IgM-positive and low/moderate HCMV IgG avidity results as well as positive DNAemia and/or seroconversion for HCMV. Nonprimary maternal HCMV infection was diagnosed, within the first 16 weeks of gestation, according to blot-confirmed IgM-positivity with high avidity anti-HCMV IgG and presence of DNA-HCMV in blood and/or urine and/or saliva.

HCMV-seronegative women (for both IgG and IgM) were defined as uninfected.

Fifty-two nonpregnant women with HCMV past infection (IgG positive and IgM negative) were recruited as healthy controls.

Moreover, 56 amniotic fluid samples were collected during amniocentesis (20-21 weeks of gestation) from those pregnant women with primary HCMV infection arising before the 14th week of gestation; HCMV detection on amniotic fluid samples was performed with virus isolation and real-time PCR.

### 2.2. Diagnosis of Congenital HCMV Infection in Fetuses and Infants

Infection status of the aborted fetuses was classified on the basis of histological and immunohistochemical tissue examination, whereas the infection status of infants was classified on the basis of viral isolation and real-time PCR from urine within the first 2 weeks of life.

Fetal symptomatic infection was defined as the presence of ultrasound abnormalities and histological and immunohistochemical findings in fetal organs with particular attention to the brain [21]. CMV disease in infected newborns was investigated through clinical, instrumental, and laboratory examination in the neonatal and subsequently monitored up to 6 years of age [22].

### 2.3. Anti-HCMV IgM and IgG Detection and IgG Avidity

Maternal serum samples were tested using the Enzygnost® HCMV IgM and Enzygnost HCMV IgG assays (Siemens Healthcare Diagnostics) and an in-house immunoblot for detection of HCMV-specific IgM [23]. HCMV IgG avidity was tested with the Radim® Cytomegalovirus IgG Avidity EIA WELL assay (Radim).

### 2.4. Virological Examinations

HCMV isolation from amniotic fluid was performed by shell-vial procedure as described elsewhere [24]. DNA was extracted from amniotic fluid and saliva with the NucliSens easyMAG System (bioMerieux) and from blood and urine with the QIAsymphony SP/AS System (QIAGEN).

HCMV-DNA was quantified with a real-time PCR assay (HCMV ELITe MGB kit, ELITechGroup) using the ELITe MGB technology. Amplification, detection, and analysis were performed with the ABI PRISM 7300 platform (PE Applied Biosystems). The detection limit was 11 copies/reaction and viral load was reported as number of copies/mL for all body fluids examined.

### 2.5. Enzyme-Linked Immunosorbent Assay (ELISA) for Soluble HLA-G

sHLA-G levels in serum and amniotic fluid samples were assayed in triplicate as previously reported [25, 26] using the monoclonal antibody (MoAb) MEM-G9 (Exbio), which recognizes the HLA-G molecules, in b2M associated form. The intra-assay coefficient of variation (CV) was 1.4% and the interassay CV was 4.0%. The limit of sensitivity was 1.0 ng/mL.

### 2.6. ELISA for Soluble Beta-2 Microglobulin and Albumin

b2M concentration was determined in triplicate using a commercial human beta-2 microglobulin ELISA Kit (Abcam) with a detection limit <6 pg/mL.

Albumin concentration was determined in triplicate with a 1 : 200 dilution using the commercial human albumin ELISA Kit (Alpha Diagnostic International) with intra-assay CV of 6.8 to 11.4% and interassay CV of 3.5 to 6.4%.

### 2.7. Determination of sHLA-G Index

Fetal production of HLA-G was calculated with the following formula [27]:

(1) $$\begin{array}{c} \text{sHLA-G Index}=\frac{\text{amniotic fluid : serum sHLA-G}}{\text{amniotic fluid : serum albumin}}, \end{array}$$

where the ratio between amniotic fluid and serum albumin concentrations represents the status of placental barrier.

### 2.8. Western Blot Analysis

Serum samples and amniotic fluids were biotinylated with 0.2 mg/mL EZ-Link Sulfo-NHS-LC-Biotin (Pierce) in pH 8.0 PBS 1x for 30 min at 4°C [26]. Samples were then immunoprecipitated for 2 hrs at room temperature with anti-HLA-G MoAb (MEM-G1, specific for HLA-G free-heavy chain, or MEM-G9, specific for b2M conjugated HLA-G, Exbio), washed twice in PBS 1x, and incubated overnight with protein G-Sepharose beads (Santa Cruz) at 4°C. The samples were washed twice and resuspended in 20 *μ*L Laemmli Buffer (BioRad). We quantified protein concentration in immunoprecipitates by the Bradford assay (Bio-Rad Laboratories) using bovine albumin (Sigma-Aldrich) as standard. Total protein was denatured at 100°C for 5 min. Proteins were loaded with native or reducing buffers in 10% TGX-Precast gel (Biorad), with subsequent electroblotting transfer onto a PVDF membrane (Millipore) [28]. The membrane was incubated with a horseradish peroxidase- (HRP-) conjugated antimouse antibody (1 : 5000; Amersham Biosciences) and developed with the ECL kit (Amersham Biosciences). The images were acquired by Geliance 600 (Perkin Elmer).

### 2.9. Statistics

Statistical analysis was performed with Stat View software package (SAS Institute Inc). Given that the data, screened by Kolmogorov-Smirnov test, presented a normal distribution, statistical analyses were performed using Student's *t*-test. Frequencies of positive samples for a specific variable were compared by Fisher exact test. A logistic regression analysis was performed to evaluate the effect of different variables. The relationship between sHLA-G presence and HCMV infection status was investigated by the Receiver Operating Characteristic (ROC) curve analysis (JROCFIT software, John Hopkins University).

## 3. Results

### 3.1. sHLA-G and Beta-2 Microglobulin Levels in Maternal Serum Samples

We evaluated sHLA-G and b2M levels in sera of 130 pregnant women, 30 uninfected, 56 with primary HCMV infection, and 44 with nonprimary HCMV infection, and 52 nonpregnant women with HCMV past infection.

Detectable serum levels of sHLA-G were significantly more frequent in pregnant (130/130; 100%) than in nonpregnant women (31/52; 59.6%) (*p* < 0.0001). b2M molecules presented only slightly higher positive samples in pregnant women (65/100; 50%) than in nonpregnant women (20/52; 38.5%) (*p* = 0.14) (data not shown).

**Figure 1:**
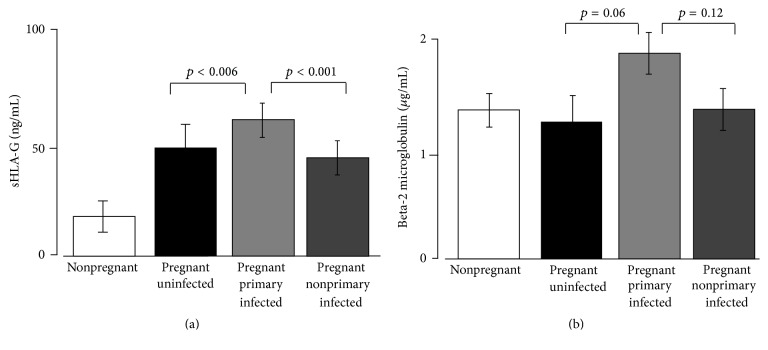
Maternal serum samples expression of (a) sHLA-G and (b) b2M molecules in nonpregnant and pregnant women. Pregnant women are classified as uninfected and primarily and nonprimarily HCMV infected. *p* values obtained by Student's *t*-test and mean ± standard deviation are reported.

Regarding the median levels of sHLA-G, pregnant women showed higher levels of molecules in comparison with nonpregnant women (49 versus 21 ng/mL; range: 37.4–76.5 ng/mL versus 0.0–20.5 ng/mL) irrespective of HCMV infection (*p* < 0.001) (Figure 1(a)). In addition, we observed no statistical differences in sHLA-G serum median levels between actively HCMV infected (56 with primary and 44 with nonprimary infection) and 30 uninfected pregnant women (47 versus 50 ng/mL; range: 36.2–69.8 versus 37.4–76.5 ng/mL; *p* = 0.43) (data not shown).

Interestingly, subdividing the subjects according to the maternal HCMV infection status, primary infected pregnant women presented higher levels of sHLA-G median concentrations (62 ng/mL; 46.9–69.8 ng/mL) than nonprimary infected women (44 ng/mL; 36.2–57.2 ng/mL) (*p* < 0.001). Moreover, primary infected pregnant women presented higher sHLA-G median concentrations than uninfected women (50 ng/mL; 37.4–76.5 ng/mL) (*p* = 0.006) (Figure 1(a)).

The median levels of b2M were only slightly higher in actively HCMV infected (primary and nonprimary) than in uninfected pregnant women (1.8 versus 1.3 *μ*g/mL; *p* = 0.14), in primary than in nonprimary HCMV infected (1.9 versus 1.4 *μ*g/mL; *p* = 0.12), and, finally, in primary HCMV infected than in uninfected women (1.9 versus 1.3 *μ*g/mL; *p* = 0.06) (Figure 1(b)).

### 3.2. sHLA-G and Beta-2 Microglobulin Levels in Amniotic Fluid

We evaluated sHLA-G and b2M levels in 56 amniotic fluid samples from women who were primarily HCMV infected before week 14 of gestation and accepted amniocentesis.

Out of the 56 amniotic fluids, 39 samples were HCMV negative with PCR and virus isolation and no congenitally infected newborns were found in this group.

Out of 17 amniotic fluids from mothers who transmitted the virus to their fetuses/babies, two were negative for both virus isolation and PCR. Despite these negative results, the 2 babies were congenitally infected, but asymptomatic at birth and during the follow-up period, as already described in the literature [9, 10]. The remaining 15 amniotic samples were positive for both virological tests (6 × 10^5^ copies/mL median viral load) except for one case where only HCMV-DNA was detected (10^3^ copies/mL).

Overall, out of the 17 fetuses/babies infected with congenital HCMV, 5 newborns were asymptomatic at birth and during subsequent monitoring and 11 fetuses and 1 newborn were symptomatic.

The brains of all symptomatic fetuses were HCMV positive with severe histological brain damage and cerebral necrosis; 4 of the fetuses also showed pathological neurosonographic findings (periventricular hyperechogenicity and ventriculomegaly).

Moreover, the only symptomatic newborn had hepatosplenomegaly, thrombocytopenia (platelet count: <100.000/mm^3^), and alanine aminotransferase elevation (>80 U/L) at birth and developed sequelae with sensorineural hearing loss and mild psychomotor retardation.

No statically significant difference in amniotic fluid HCMV load was observed between asymptomatic and symptomatic fetuses/newborns (*p* = 0.88, 95% CI: −3583021 to 3110188).

All the 56 amniotic fluid samples were positive for sHLA-G and b2M molecules (data not shown).

**Figure 2:**
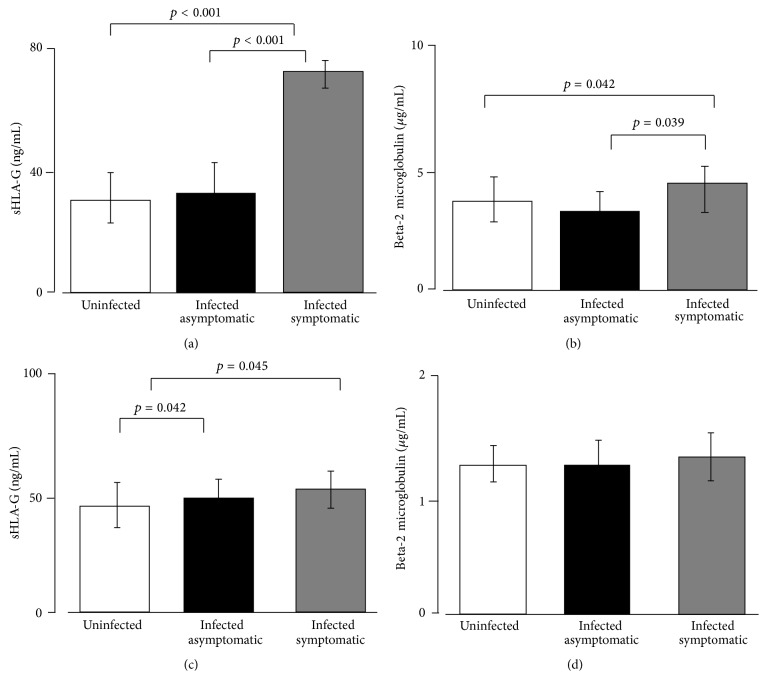
Amniotic fluid samples expression of (a) sHLA-G and (b) b2M molecules according to the fetal/neonatal outcome. Maternal serum samples expression of (c) sHLA-G and (d) b2M molecules according to the fetal/neonatal outcome. *p* values obtained by Student's *t*-test and mean ± standard deviation are reported.

Median detectable levels of sHLA-G were significantly higher in amniotic fluids from infected symptomatic fetuses (73 ng/mL; 69–79 ng/mL) than in infected asymptomatic fetuses (32 ng/mL; 28–42 ng/mL) (*p* < 0.001) and in uninfected fetuses (31 ng/mL; 29–40.2 ng/mL) (*p* < 0.001) (Figure 2(a)).

b2M presented slightly higher median levels in amniotic fluids from infected symptomatic fetuses (4.5 *μ*g/mL) than in infected asymptomatic fetuses (3.6 *μ*g/mL) (*p* = 0.039) and uninfected fetuses (3.9 *μ*g/mL) (*p* = 0.042) (Figure 2(b)).

When we considered maternal serum levels according to fetus infection status, we observed that sHLA-G concentrations were slightly higher in serum from women with symptomatic fetuses (51.2 ng/mL, 45–57 ng/mL) and in women with infected asymptomatic fetuses (49 ng/mL, 42–55 ng/mL) in comparison with uninfected fetuses (46 ng/mL, 44–52 ng/mL) (*p* = 0.045, *p* = 0.042, resp.) (Figure 2(c)).

b2M presented no differences in serum samples from women with infected symptomatic fetuses, infected asymptomatic fetuses, and uninfected fetuses (Figure 2(d)).

### 3.3. sHLA-G Concentration Gradient between Maternal Serum and Amniotic Fluid

The sHLA-G increase in amniotic fluids of infected symptomatic fetuses prompted the question of whether sHLA-G was produced locally in amniotic compartment or derived from maternal blood. Fetal and maternal compartments are mutually interconnected and several molecules are exchanged through the amniotic and chorionic membrane. This molecular interchange could be hypothesized also for HLA molecules. Therefore we evaluated the concentration gradient between serum samples from primary infected women and the corresponding amniotic fluids.

**Figure 3:**
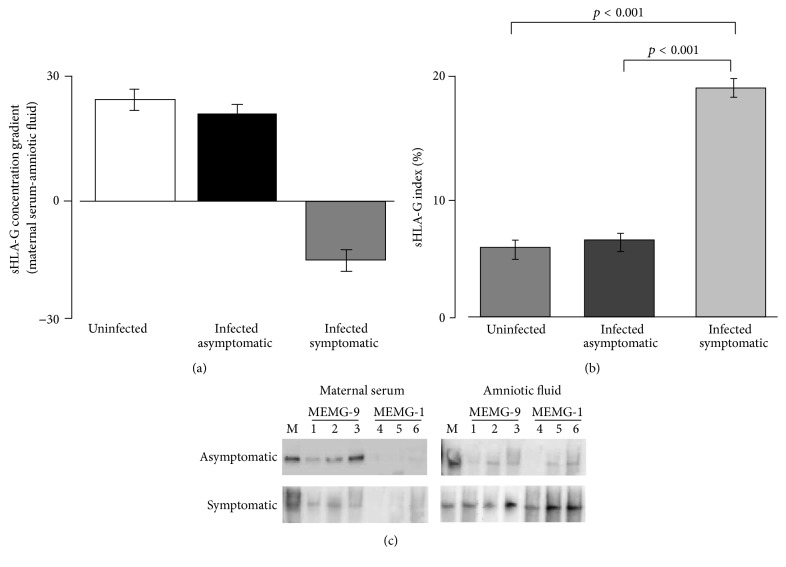
(a) sHLA-G concentration gradients according to the fetal/neonatal outcome. (b) sHLA-G indexes (%) according to the fetal/neonatal outcome. (c) Representative Western blot analysis of maternal serum and amniotic fluids samples from HCMV primary infected pregnancy subdivided according to the fetal/neonatal outcome. The analysis were performed after immunoprecipitation with anti-b2M associated HLA-G moAb (MEM-G9, Exbio) (lines 1 to 3) and anti-free HLA-G HC moAb (MEMG-1, Exbio) (lines 4 to 6). JEG3 cell line supernatants were used as positive control (M) and the positivity for HLA-G molecule was evidenced at 39 kD.

A sHLA-G concentration gradient from the amniotic fluid to the maternal serum was observed only in infected symptomatic fetuses, while uninfected and infected asymptomatic fetuses presented an inverse sHLA-G gradient (Figure 3(a)). These results suggest a local fetal production of sHLA-G, increased only in fetuses with symptomatic HCMV infection.

### 3.4. sHLA-G Index

The association between fetal HCMV infection and increased sHLA-G expression in amniotic fluid was confirmed calculating the sHLA-G index in comparison with albumin. Albumin is the most prevalent serum protein which surrounds the embryo and is detected in amniotic fluids. Fainardi et al. [27] reported the use of cerebrospinal fluid and serum albumin content to evaluate sHLA-G brain production. Since both blood-brain interface and placenta are considered selective barriers, we applied the same concept to quantify the fetal compartment production of sHLA-G, evaluating the relative amount of amniotic sHLA-G and albumin compared with maternal serum levels. Any increase in the index could be ascribed to sHLA-G production in the fetal compartment. The highest sHLA-G indexes were detected in infected symptomatic fetuses (19.5%) compared to infected asymptomatic fetuses (6%) and uninfected (5.1%) (*p* < 0.001, *p* < 0.001, resp.) (Figure 3(b)).

### 3.5. HLA-G Free-Heavy Chain Analysis

HLA-G can be expressed as b2M associated or free-heavy chain. Previous studies documented a different distribution of these two conformations at the maternal-fetus interface [29]. We evaluated the presence of HLA-G free-heavy chain in both sera and in amniotic fluids from primary HCMV infected pregnant women with asymptomatic or symptomatic fetus. HLA-G free-heavy chain was detected with a tendency to be more frequent in amniotic fluids from symptomatic fetuses (*p* = 0.074). On the contrary, maternal sera did not present HLA-G free-heavy chain (Table 1).

Representative examples of maternal serum and AF reactivity to antigens on the Western blot are shown in Figure 3(c). In lines 1, 2, and 3, we reported HLA-G positive samples analyzed for b2M associated form (MEM-G9 detection), while the same samples were analyzed for HLA-G free-heavy chain (MEM-G1 detection) in lines 4, 5, and 6.

### 3.6. sHLA-G Predictive Efficacy

We analyzed serum and amniotic fluid samples for sHLA-G and selected different cut-offs to be used as differentiation values. ROC analysis showed serum values above 50 ng/mL and amniotic values above 30 ng/mL with the highest sensitivity and specificity and an Area Under an ROC Curve of 0.83 and 0.86, respectively (Table 1), in differentiating symptomatic from asymptomatic congenital infections. Similarly, the presence of HLA-G free-heavy chain in amniotic fluids shows a high sensitivity and specificity and an Area Under an ROC Curve of 0.79, in identifying symptomatic congenital infections. Logistic regression analysis excluded the presence of confounding variables.

## 4. Discussion

The prenatal diagnosis provides the optimal means for diagnosing HCMV fetal infection. The specificity is very good and the sensitivity depends on the kind of samples used (amniotic fluid > fetal blood), the technique used (Real-Time PCR-Polymerase Chain Reaction > viral culture), and the timing of the procedure with respect to the onset of maternal infection and the gestational age.

All literature data report that the amniotic fluid is the most appropriate material for the diagnosis of fetal HCMV infection. Positive results in amniotic fluid identify all HCMV infected fetuses (positive predictive value = 100%) but do not identify the infants who will have symptoms at birth [6].

Although the highest median values of HCMV-DNA in amniotic fluid tend to indicate an increased risk of severe infection, high viral loads may be associated with symptomatic or asymptomatic congenital infections. Indeed, a correlation between the high HCMV load in amniotic fluid and fetal/neonatal outcome has not been demonstrated [6].

More recently, some studies have evoked the prognostic value of fetal viremia/viral load and/or level of specific IgM; however this remains controversial. It has been proposed that platelet count gives a better indication. New data has demonstrated that the determination of multiple markers (haematological, biochemical, and virological markers) in fetal blood following virus detection in amniotic fluid is predictive of perinatal outcome in fetuses with HCMV infection [21]. Further studies in a larger number of symptomatic cases should be performed to verify the prognostic efficacy of determination of multiple parameters in fetal blood.

In this work, we analyzed the levels of sHLA-G and b2M molecules in the maternal serum samples and showed that sHLA-G median levels were significantly higher in maternal serum from pregnant women with primary HCMV infection than in nonprimary and uninfected, respectively (*p* < 0.001 and *p* < 0.006). On the contrary, beta-2 microglobulin levels were only slightly higher in maternal serum from pregnant women with primary HCMV infection than in nonprimary and uninfected, respectively (*p* = 0.12 and *p* = 0.06). The differences in immune competence towards HCMV in primary infected and in nonprimary infected mothers could explain the different production of sHLA-G that can act as viral immune escape mechanism or as a tentative reduction of immune activation in primary HCMV infection, which is not induced in nonprimary HCMV infected women.

Furthermore, we evaluated whether sHLA-G molecules could be considered prognostic biomarkers of symptomatic congenital HCMV infection.

We observed that sHLA-G levels in both maternal serum and amniotic fluid samples are significantly related to symptomatic HCMV fetal infections as supported by ROC analysis (Table 1). In fact, sHLA-G levels above 50 ng/mL in maternal blood and 30 ng/mL in amniotic fluid are correlated with symptomatic HCMV congenital infections. High levels of sHLA-G in maternal and fetal compartments show a specificity of 100% for symptomatic congenital infection and a sensitivity ranging between 83 and 92%.

The evidence that sHLA-G levels increase only in the presence of symptomatic fetuses suggests a specific fetal production of these molecules. We obtained confirmation of this hypothesis through (i) the evaluation of the concentration gradient, which is higher in the amniotic fluid versus the maternal blood in case of symptomatic infection; (ii) the sHLA-G indexes calculation, which support a fetal production; (iii) the HLA-G free-heavy chian, which is commonly expressed by distal trophoblasts [29], which is present only in amniotic fluid.

The increase of fetal HLA-G expression could be caused by HCMV-encoded proteins that are known to interact with HLA mRNAs and proteins, modifying their stability and secretory pathways [15–19]. The increase of HLA-G expression could enhance HCMV immune escape, increasing the risk of congenital infections and symptomatic sequelae. Our results suggest that serum and amniotic fluid sHLA-G might be an additional biomarker of congenital HCMV infection that could be considered in combination with currently used viral and biological markers [20, 30], providing the best key to the reliable identification of fetuses at risk of congenital disease as well as of fetuses with a favorable outcome.

## 5. Conclusions

To the best of our knowledge, this is the first observation that considers the possible use of serum and amniotic fluid sHLA-G as a biomarker to discriminate between symptomatic and asymptomatic HCMV congenital infection. However, future studies with larger cohort of fetuses should be performed in order to verify whether the addition of serum sHLA-G determination to virologic markers may be crucial in identifying fetuses at highest risk of severe pathologies.

## Figures and Tables

**Table 1:** Prognostic HLA-G biomarker of symptomatic congenital HCMV infection.

Parameters	Cutoff		Fetuses		Diagnostic accuracy				AUA
					95% CI				
			Sympt	Asympt	Sens%	Spec%	PPV%	NPV%	
*Maternal serum samples*									
HLA-G free-heavy chain		Presence	0	0	0	100	0	29.4	0.5
		Absence	12	5	0–26.7	47.9–100		10.4–55.9	
sHLA-G	50 ng/mL	Above	10	0	83.3	100	100	71.4	0.83
		Below	2	5	51.6–97.4	47.9–100	68.9–100	29.3–95.5	

*Amniotic fluid samples*									
HLA-G free-heavy chain		Presence	12	3	100	40	80	100	0.79
		Absence	0	2	73.3–100	6.4–84.6	51.9–95.4	19.2–100	
sHLA-G	30 ng/mL	Above	11	0	91.7	100	100	83.3	0.86
		Below	1	5	61.5–98.6	47.9–100	71.3–100	36.1–97.2	

Sens: sensitivity; Spec: specificity; PPV: positive predictive value; NPV: negative predictive value; AUA: Area Under an ROC Curve; Sympt: symptomatic; Asympt: asymptomatic.
